# Alcohol-Related Morbidity and Mortality

**Published:** 2003

**Authors:** Jürgen Rehm, Gerhard Gmel, Christopher T. Sempos, Maurizio Trevisan

**Affiliations:** Jürgen Rehm, Ph.D., is chief executive officer and director of the Addiction Research Institute, Zurich, Switzerland; professor in the Department of Public Health Sciences, University of Toronto; and senior scientist at the Centre for Addiction and Mental Health, Toronto, Canada. Gerhard Gmel, Ph.D., is a co-director of research at the Swiss Institute for the Prevention of Alcohol and Drug Problems, Lausanne, Switzerland. Christopher T. Sempos, Ph.D., is a professor and director of graduate studies, and Maurizio Trevisan, M.D., M.S., is a professor and chairman in the Department of Social and Preventive Medicine, School of Medicine and Biomedical Sciences, both at the University at Buffalo, Buffalo, New York

**Keywords:** AODR (alcohol and other drug related) mortality, morbidity, epidemiological indicators, chronic AODE (alcohol and other drug effects), acute AODE, amount of AOD use, alcoholic beverage, heart disorder, meal and meal time

## Abstract

Alcohol use is related to a wide variety of negative health outcomes including morbidity, mortality, and disability. Research on alcohol-related morbidity and mortality takes into account the varying effects of overall alcohol consumption and drinking patterns. The results from this epidemiological research indicate that alcohol use increases the risk for many chronic health consequences (e.g., diseases) and acute consequences (e.g., traffic crashes), but a certain pattern of regular light-to-moderate drinking may have beneficial effects on coronary heart disease. Several issues are relevant to the methodology of studies of alcohol-related morbidity and mortality, including the measurement of both alcohol consumption and the outcomes studied as well as study design. Broad summary measures that reflect alcohol’s possible effects on morbidity, mortality, and disability may be more useful than measures of any one outcome alone.

Alcohol use contributes to a range of acute and chronic health consequences, from injuries resulting from traffic crashes to cancer and cardiovascular disease. Research has explored the relationships between the risk for alcohol-related morbidity and mortality and both the overall amount of alcohol consumed and the pattern of drinking. This article will review this research, with a focus on the relationship between alcohol use and coronary heart disease (CHD).

Alcohol-related mortality is studied more frequently than alcohol-related morbidity. More than 80 studies have examined the relationship between a person’s average volume of alcohol consumption (i.e., average number of drinks per day) and alcohol-related mortality (see Rehm et al. 2001b for a meta-analysis of studies conducted through 1999). Research has linked varying average levels of alcohol consumption (i.e., light, moderate, heavy) to increased and sometimes decreased risk for morbidity and mortality related to more than 60 disease conditions (English et al. 1995; Single et al. 1999; Gutjahr et al. 2001; Ridolfo and Stevenson 2001; Rehm et al. in press *b* ). Some of these research findings are reflected in the table accompanying this article.

Fewer studies examine alcohol-related morbidity alone or a combination of morbidity and mortality. One study that grouped morbidity and mortality together examined the impact of alcohol on coronary heart disease (CHD)(Rehm et al. 1997); in this study, which used data from the National Health and Nutrition Examination Epidemiologic Follow-Up Study, based on a large representative survey of the U.S. general population, the data did not distinguish between people newly diagnosed with CHD and people who had died of the disorder. Overall, information about alcohol-related morbidity alone is limited because studies with morbidity as the endpoint demand substantial resources to assess individual outcomes in an objective and standardized way.

Even scarcer than studies of alcohol-related morbidity are studies of the effects of alcohol consumption on disability or quality of life (i.e., how alcohol use causes health-related activity limitations, as defined in the *International Classification of Functioning, Disability and Health* (World Health Organization [WHO] 2001). The lack of studies linking alcohol use to disability or quality of life is on the one hand surprising, as the first global study of alcohol-related morbidity and mortality clearly indicated that alcohol causes a larger proportion of global disability than global mortality. Specifically, it found that 1.5 percent of all deaths were attributable to alcohol, but 6 percent of all life years lost to disability were attributable to alcohol (Murray and Lopez 1996). On the other hand, even in developed countries, investigators do not collect as much data on disability as they do on mortality, because mortality is easier to quantify and data recording is required by law (i.e., a death certificate must be filled out in a standardized way) (Goerdt et al. 1996; see also Rehm and Gmel 2000). Unlike the registration of deaths, there is no routine registration of disability, which would allow relatively easy access for research purposes, linking other data such as alcohol use to disability endpoints. That is, if a disability registration existed, researchers could more easily study the link between alcohol use and disability. As a result, studies on disability are harder to conduct and require more resources. Despite these challenges, disability and quality of life have been receiving increasing attention as health outcomes, both subjectively and as part of summary measures of health (i.e., measures that integrate effects on morbidity, mortality, and disability) (Murray et al. 2000).

The following sections examine the possible chronic and acute health consequences of alcohol use, focusing on the example of CHD. This review does not examine alcohol’s role in the social, legal, and financial consequences of alcohol use and alcohol-related injury, as this subject is covered elsewhere (see Rehm 2001; Klingemann and Gmel 2001; see also the article by Gmel and Rehm in this issue).

## Chronic Consequences of Alcohol Use

The table gives an overview of the risks for major chronic diseases related to varying levels of alcohol consumption, based on the results of observational data from cohort and case control studies that mainly used mortality as an endpoint. Because data on morbidity alone are not sufficiently available for most disease conditions (Single et al. 1999), meta-analyses usually combine mortality and morbidity as endpoints. In cohort studies, researchers evaluate a group of people, known as a cohort, who are disease free at the beginning of the study, to assess if they have been exposed to potential risk factors. The cohort is then followed over time and monitored for the occurrence of disease endpoints and, in some studies, changes in risk factor exposure. The objective is to assess which risk factors are related to the risk of developing a disease or condition. The purpose of case control studies is to assess whether people affected by a disease (i.e., cases) are more or less likely than a comparable group of people who do not have the disease (i.e., control subjects) to have been exposed to the relevant risk factors before developing the disease (see Gordis 1999).

In the table, relative risk estimates are shown to quantify the effect size of the risk relationships. For example, females in drinking category I, who drink on average up to 20 grams of pure alcohol per day,^1^ have a relative risk of 1.14, compared with female abstainers, of developing breast cancer. A relative risk of 1.14 corresponds to a 14-percent higher risk. For females drinking more than 40 grams of pure alcohol per day (drinking category III), the relative risk is 1.59, or about one and one-half times as large as for female abstainers, and corresponds to a 59-percent risk increase.

The International Classification of Diseases (ICD) is a system for coding both nonfatal and fatal events. Under the ICD system, the medical information provided on the death certificate, for example, is coded to indicate the underlying cause of death. The table shows the ICD codes from the 9th and 10th editions of the ICD for each disease group (WHO 1977, 1992).

As shown in the table, research has demonstrated that, for many chronic diseases, the risk of disease increases with increasing average daily alcohol consumption. Most of the major disease categories listed in the table have not been linked to specific patterns of drinking. However, this may be the result of the lack of investigation into such relationships. For example, although researchers have speculated that breast cancer risk may be influenced by the frequency of heavy drinking episodes (defined as the consumption of more than five drinks on one occasion without regularly drinking this amount), no research has explored this relationship (Kohlmeier and Mendez 1997). One disease risk that is clearly affected by the pattern of alcohol consumption is the risk for cardiovascular disease (CVD), especially CHD (see below).

**Table:** Relative Risk for Major Chronic Disease Categories, by Gender and Average Drinking Category

Disease			Females			Males		
	
	ICD–9 code	ICD–10 code	Drinking Category*					

			I	II	III	I	II	III

**Malignant neoplasms**	140–208	C00–C97						

Mouth and oropharynx cancers	140–149	C00–C14	1.45	1.85	5.39	1.45	1.85	5.39

Esophagus cancer	150	C15	1.80	2.38	4.36	1.80	2.38	4.36

Liver cancer	155	C22	1.45	3.03	3.60	1.45	3.03	3.60

Breast cancer			1.14	1.41	1.59			
Under 45 years of age	174	C50	1.15	1.41	1.46			
45 years and over			1.14	1.38	1.62			

Other neoplasms	210–239	D00–D48	1.10	1.30	1.70	1.10	1.30	1.70

Diabetes mellitus	250	E10–E14	0.92	0.87	1.13	1.00	0.57	0.73

**Neuropsychiatric conditions**	290–319, 324–359	F01–F99, G06–G98						

Unipolar major depression	300.4	F32–F33	RR not available; AF could not be determined otherwise (Rehm et al., in press *b*)					

Epilepsy	345	G40–G41	1.34	7.22	7.52	1.23	7.52	6.83

Alcohol use disorders	291, 303, 305.0	F10	AF** 100%†	AF 100%	AF 100%	AF 100%	AF 100%	AF 100%

**Cardiovascular diseases (CVD)**	390–459	I00–I99						

Hypertensive disease	401–405	I10–I13	1.40	2.00	2.00	1.40	2.00	4.10

Coronary heart disease	410–414	I20–I25	0.82	0.83	1.12	0.82	0.83	1.00

Cerebrovascular disease	430–438	I60–I69						

Ischemic stroke			0.52	0.64	1.06	0.94	1.33	1.65

Hemorrhagic stroke			0.59	0.65	7.98	1.27	2.19	2.38

Other CVD causes	415–417, 423– 424, 426–429, 440–448, 451– 459	I00, I26–I28, I34–I37, I44– I51, I70–I99	1.50	2.20	2.20	1.50	2.20	2.20

**Digestive diseases**	530–579	K20–K92						

Cirrhosis of the liver	571	K70, K74	1.26	9.54†	9.54†	1.26	9.54†	9.54†

NOTE: Relative risk estimates are shown to quantify the effect size of the risk relationships. For example, females in drinking category I have a relative risk of 1.14, compared with female abstainers, of breast cancer. A relative risk of 1.14 corresponds to a 14-percent higher risk. For females in drinking category III, the relative risk is 1.59, or about one and one-half times as large as for female abstainers. The same relationship can also be expressed as a risk increase of 59 percent.

Varying numbers of studies were used to report on the different diseases. Measurement problems for outcomes affected the reliability of the data for some endpoints, especially the different subtypes of strokes and the unspecified categories such as “other cardiovascular disease” or “other neoplasms.” The results for these categories should be regarded with caution.

* Definition of drinking categories:
Category I: for females, 0–19.99 g pure alcohol daily; for males, 0–39.99 g pure alcohol daily Category II: for females, 20–39.99 g pure alcohol daily; for males, 40–59.99 g pure alcohol daily Category III: for females, 40 g or more pure alcohol; for males, 60 g or more pure alcohol.

** AF = attributable fraction—that is, the proportion of disease under consideration that is attributable to alcohol.

† For liver cirrhosis, a combined estimate was derived for drinking categories II and III.

SOURCES: Unless otherwise specified, Gutjahr et al. 2001; for breast cancer and stroke, Ridolfo and Stevenson 2001; for hypertension, Corrao et al. 1999; for CHD, drinking category III, Corrao et al. 2000.

### Alcohol Consumption, Coronary Heart Disease, and Other Cardiovascular Outcomes

#### Average Light-to-Moderate Drinking

The most comprehensive meta-analysis on average consumption and CHD found that this relationship was represented by a J-shaped curve (see the accompanying figure) (Corrao et al. 2000). That is, compared with abstinence from alcohol, low-to-moderate average consumption of alcohol is associated with lower risk for CHD incidence and mortality, the lowest risk being found at 20 grams per day. For higher levels of average volume of alcohol consumption, the risk relationship reverses (Corrao et al. 2000; Rehm et al. 1997), with average consumption of more than 70 grams per day associated with greater risk than the risks for abstainers. Several physiological mechanisms have been suggested to explain the protective effect of moderate drinking, including alcohol’s role in reducing plaque deposits in arteries and the fact that moderate alcohol consumption protects against blood clot formation and promotes blood clot dissolution (Zakhari 1997; Rehm et al. 2003, in press *b*). However, most of these mechanisms are thought to apply only for cohorts in which a majority of respondents have reported a pattern of regular drinking without variability. As most of the studies analyzed by Corrao and colleagues (2000) used cohorts formed by groups with such patterns, further research is needed to determine how patterns of drinking other than regular light-to-moderate drinking are linked to CHD, especially among cohorts of usually light-to-moderate drinkers who sometimes binge drink.

**Figure:**
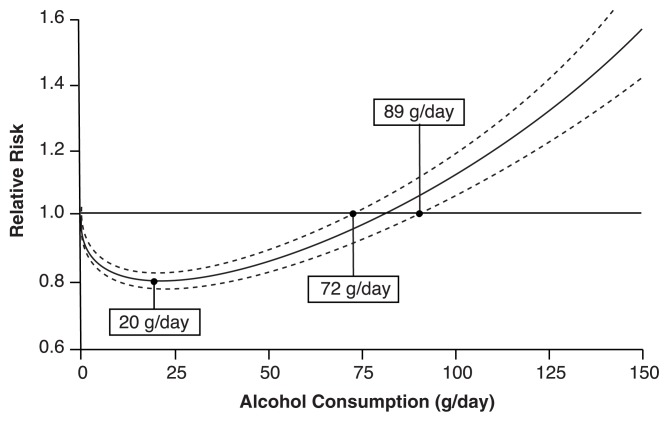
Example of relationship between average alcohol consumption and CHD, as expressed by a J-shaped curve with confidence intervals. NOTE: The middle line represents the result of the meta-analysis; the other two represent the lower and upper confidence intervals. SOURCE: Corrao et al. 2000.

#### Binge Drinking and CHD

A case control study in Australia (McElduff and Dobson 1997) compared 11,511 nonfatal and fatal cases of acute myocardial infarction, or coronary death, with 6,077 randomly selected people from the general population (i.e., control subjects). This study confirmed the already mentioned protective effect of moderate drinking, which was most pronounced for regular light-to-moderate drinkers. For example, men and women who drank one to two drinks per day on 5 or 6 days per week had one-third the risk of major coronary events compared with those who did not drink at all. The risk reduction was still marked when former drinkers were excluded. However, McElduff and Dobson (1997) found that binge drinkers (i.e., women who consumed five or more drinks on an occasion, or men who consumed nine or more drinks on an occasion) had higher risks for major coronary events than abstainers, even when overall volume of drinking was low. More recent studies that followed participants over time (i.e., prospective studies) also concluded that heavy drinking occasions increased the risk of CHD even in light-to-moderate drinkers (Murray et al. 2002; Trevisan et al. 2001a, b). This pattern effect persisted after controlling for average volume of drinking.

#### Binge Drinking and Other Negative Cardiovascular Effects

In addition to its effect on CHD, an irregular pattern of heavy drinking occasions appears to have a relationship with other types of cardiovascular death such as stroke or sudden cardiac death (e.g., Wannemethee and Shaper 1992; Kauhanen et al. 1997). This relationship is consistent with the increased clotting and lowered threshold for ventricular fibrillation that occur after heavy drinking (see reviews by McKee and Britton 1998 and Rehm et al. 2003). (Heavy drinking appears to lower the threshold at which the ventricular heart muscle begins a rapid contraction pattern; without prompt intervention, this pattern prevents normal heart function and results in death.) Specifically, heavy drinking occasions have been shown to increase low density lipoproteins, which have been linked to negative cardiovascular outcomes. Although regular low-to-moderate drinking is associated with an increase in high density lipoproteins, which have been linked to favorable cardiovascular outcomes, this effect is not associated with irregular heavy drinking occasions (for a meta-analysis of the effect of moderate drinking on lipids and other physiological outcomes, see Rimm et al. 1999). In addition, irregular heavy drinking is associated with increased risk for the formation of blood clots within blood vessels (i.e., thrombosis), which occurs at the end of a heavy drinking occasion (Renaud and Ruf 1996). Finally, irregular heavy drinkers seem predisposed to structural (i.e., histological) changes in the heart muscle and the adjacent impulse-conducting system, which regulate the threshold for ventricular fibrillation. In sum, a pattern of irregular heavy drinking occasions is mainly associated with physiological mechanisms that increase the risk of sudden cardiac death and other cardiovascular outcomes, whereas regular low-to-moderate alcohol consumption is associated with physiological mechanisms linked to favorable cardiac outcomes (for overviews of the effects of drinking pattern on CVD, see Puddey et al. 1999; Rehm et al. 2003). However, individual-level epidemiological studies on the consequences of drinking (i.e., studies, such as cohort and case control studies, which evaluate groups of individuals rather than entire populations) are still scarce, and at least one of them has found heavy drinking occasions to have no detrimental effects on morbidity (Murray et al. 1998).

#### Aggregate-Level Studies

Because there are few individual-level studies, much of the discussion on patterns of drinking and outcomes is based on aggregate-level studies, which evaluate whole populations rather than individual study participants. Much of this research emerged from the natural experiment provided by the Gorbachev anti-alcohol campaign in what was then the Soviet Union. In this campaign, all government departments were asked to develop strategies to reduce alcohol consumption, which was seen as hindering the country’s development. Numerous measures were implemented to limit access to alcohol, including banning alcohol at the workplace, limiting alcohol sales hours, restricting the number of alcohol outlets, reducing alcohol production, and increasing prices by 25 percent in 1985 alone. In addition, the All-Union Voluntary Society for the Struggle for Sobriety was created in September 1985 to raise public awareness and mobilize the population (McKee 1999). During the period from 1984 to 1987, when estimated total alcohol consumption in the Soviet Union fell by about 25 percent (Shkolnikov and Nemtsov 1997), age-adjusted male deaths from circulatory disease fell by 9 percent (Leon et al. 1997). Because the Soviets did not use the ICD coding system, the term “circulatory disease” is used to refer to a category roughly equivalent to CVD (see Notzon et al. 1998 for a rigorous attempt to equate the two systems). After the end of the campaign, alcohol consumption increased by about 36 percent (from 1987 to 1993) and the circulatory disease death rate rose by 29 percent (between 1987 and 1994) (Leon et al. 1997).

Although researchers agree that changes in alcohol consumption and heavy drinking occasions played a role in the changes in circulatory mortality rates in the Soviet Union, the degree of alcohol’s involvement is still questioned, as many other social changes occurred in the late 1980s and early 1990s. For example, the collapse of the Soviet Union contributed to a scarcity of medicine for treating hypertension and other forms of heart disease and to the collapse of the medical care infrastructure in general (e.g., Bobak and Marmot 1999; Notzon et al. 1998; McKee et al. 2001).

A few other aggregate-level studies have examined the influence of population-level alcohol consumption on CHD (i.e., ecological studies). Two studies that compared per capita alcohol consumption and CHD rates at a series of points in time (i.e., time-series analyses) (Hemström 2001; Skog 1983) failed to find effects even for countries where most drinking is believed to be regular and on average moderate, such as France and Italy (but see Gmel et al. 2003*a*). Other research has found that regions (e.g., Moscow, Scotland) or groups (e.g., German working males) with a tradition of heavy drinking or binge drinking occasions on weekends show disproportionally high CHD mortality on Mondays (Willich et al. 1994; Chenet et al. 1998; Evans et al. 2000).

#### Effect of Drinking With Meals

Whether or not one drinks with meals has been found to be relevant to CHD. Trevisan and colleagues (2001*a*,*b*) found that people who drank alcohol mainly with meals and snacks had consistently lower risks for CHD than those who drank at other times, after adjustment for age, education, and volume of alcohol consumed. The mechanisms accounting for this difference in risk are still not clear, although a few have been proposed. Drinking with meals has been found to reduce blood pressure (Trevisan et al. 1987; Wu and Trevisan 2001; Foppa et al. 1999) and to positively affect lipids (Veenstra et al. 1990) and the dissolution of blood clots (Hendriks et al. 1994). In addition, the presence of food in the gastrointestinal tract may reduce alcohol absorption (Gentry 2000) or increase the rate at which alcohol is eliminated from the body (Ramchandani et al. 2001).

#### Beverage-Specific Effects

Researchers have long proposed that rates of CHD vary depending on the beverage consumed, ascribing a special beneficial effect to red wine. However, Rimm and colleagues (1996) reviewed the literature with respect to beverage-specific effects on CHD and did not find any systematic evidence that the protective effect was caused by or more prominent for any kind of beverage. Although many additional publications on this topic have appeared since their review, no consistent pattern of results has emerged. It is notable that researchers have found protective effects in Bavaria (Keil et al. 1997) and the Czech Republic (Bobak et al. 2000) similar to those effects found in Mediterranean countries. In Bavaria and the Czech Republic, beer is consumed the way wine is consumed in Mediterranean countries: regularly, on an almost daily basis, with meals. The study found that the manner of consumption was more important than the type of beverage.

The overall problem with studying beverage-specific effects is that beverage preference in most cultures is linked with other variables such as socioeconomic status and lifestyle variables (e.g., Bondy and Rehm 1998). These linkages may not be the same for different cultures, but many countries show clear covariation. For example, wine drinking in beer cultures is often associated with middle- and upper-class lifestyles. This makes it almost impossible to separate the effects of the beverages from other effects.

#### Conclusions on Alcohol and CHD

What conclusions can be reached regarding the relationship between alcohol consumption and CHD? First, the physiological, individual-level, and aggregate-level research converges to demonstrate the detrimental effect of irregular heavy drinking occasions. Both case control and cohort studies have supported this detrimental effect, and it has been corroborated by research using the natural experiment of the Gorbachev campaign. The result of irregular heavy drinking also has plausible physiological pathways.

Evidence that regular light-to-moderate drinking has a beneficial effect on the cardiovascular system comes from physiological and individual-level epidemiological studies. However, aggregate-level, time-series analyses have failed to confirm this effect. Reasons for the different results for the two kinds of analyses are unknown. The time-series analyses may have been confounded by methodological problems, including a relatively short time period (Rehm and Gmel 2001; see also Hatanaka 1996; Greene 2000; Yaffee 2000). Ecological studies also have general limitations in elucidating causality. In contrast, the individual-level studies are based on stronger methodology. The accompanying sidebar reviews a number of methodological issues relevant to studies of alcohol-related morbidity and mortality.

### Alcohol Consumption and Cancer

Many studies have reported consistent relationships between average consumption of alcohol and different types of cancer. (For examples of specific meta-analyses in the last five years in addition to the overviews of all alcohol-related conditions, see Bagnardi et al. 2001*a*,*b*; Dennis 2000; Ellison et al. 2001; Smith-Warner et al. 1998; Zeegers et al. 1999.) A recent series of meta-analyses showed that drinking on average 25 grams of pure alcohol per day was associated with significantly elevated cancer risks for the following sites: oral cavity and pharynx, esophagus, stomach, colon and rectum, liver, larynx, and female breast (Bagnardi et al. 2001*a*,*b*). However, for many cancer sites, even though there is a consistent relationship between average consumption of alcohol and risk for cancer, other criteria for determining causality are lacking. For example, for lung cancer, after adjusting for smoking, one meta-analysis showed a consistent effect with a relatively large effect size (English et al. 1995). However, because evidence for the possible biological mechanism was not conclusive and residual confounding from smoking could not be excluded, the authors excluded lung cancer from the list of diseases influenced by alcohol. A later meta-analysis showed only borderline significant effects (Bagnardi et al. 2001*a*,*b*), and the most recent review concluded that the evidence for a causal relationship was not sufficient (Bandera et al. 2001). Using consistent criteria, Rehm and colleagues (in press *a*) concluded that sufficient evidence of causality existed for the following cancer sites: oral cavity and pharynx, esophagus, liver, larynx, and female breast.

### Other Major Chronic Health Consequences of Alcohol Use

Alcohol use is also related to chronic health consequences other than CVD and cancer. Most notable are neuropsychiatric and digestive diseases. The causal role of alcohol use in alcohol use disorders (e.g., alcohol dependence, alcohol abuse) is obvious. Alcohol use disorders are responsible for a considerable burden of disease in the United States and worldwide (e.g., Michaud et al. 2001). Moreover, alcohol use may cause depression (see Rehm et al. in press *b*). The fact that alcohol causes depression in some people does not exclude the possibility that some alcohol use disorders are caused by depression, or that, for some people with comorbid depression and alcohol use disorders, there may be a third cause. On the contrary, all three causal pathways seem to exist.

The association between alcohol use and liver cirrhosis is well established (e.g., Rodés et al. 1999). Researchers have considered whether irregular heavy drinking is a specific contributing factor to cirrhosis, in addition to the well-established relationship between cirrhosis and overall volume of drinking (Kozarevic et al. 1983; Rodés et al. 1999).

## Acute Consequences of Alcohol Use

Alcohol use has been associated with increased risk of injury in a wide variety of situations including motor vehicle crashes, bicycling accidents, incidents involving pedestrians, falls, fires, injuries in sports and recreational activities, interpersonal violence, and self-inflicted injuries (Cherpitel 1992; Hingson and Howland 1987, 1993; U.S. Department of Health and Human Services [USDHHS] 1997, 2000; Martin 1982, Martin and Bachman 1997; Freedland et al. 1993; Hurst et al. 1994). Some evidence from emergency room studies and police records also suggests that the presence of alcohol in the body at the time of injury may be associated with greater severity of injury and less positive outcomes (Fuller 1995; Li et al. 1997). Overall, morbidity and mortality from traumatic injury is by far the most important health consequence of alcohol use in developed countries such as Canada or the United States (Single et al. 1999).

This section highlights research examining the relationships between acute health consequences and both average volume of alcohol consumption and drinking patterns. It focuses on unintentional injuries—specifically, traffic injuries—because most of the relevant research has been conducted in this area and because traffic crashes account for most alcohol-related unintentional injuries. For additional information on the relationship between alcohol and injuries, see the article by Gmel and Rehm in this issue.

Research has shown that risk of injury is positively related to average intake of alcohol, and that injury risk starts increasing at relatively low volumes of alcohol consumption (e.g., Cherpitel et al. 1995). Two studies of injury among adults ages 51 through 61 reported a U-shaped relationship between alcohol use and occupational injury (Zwerling et al. 1996) and traumatic deaths (Ross et al. 1990). That is, the rate of injury and death was higher among people who abstained from alcohol than among those who drank small-to-moderate amounts. The rate increased again with increasing alcohol use. This pattern may be explained by the fact that people who abstain from alcohol may have existing health problems or cognitive deficits that are, in turn, related to injury risk (Zwerling et al. 1996).

Several patterns of drinking have been related to injury risk. Frequent heavy drinking and frequent drunkenness are both associated with injury, particularly injury resulting from violence (Cherpitel 1996). Frequency of heavy drinking has also been associated with a greater likelihood of death from injury than from other causes (Li et al. 1994).

Research has also found that people who consume relatively large amounts of alcohol on some occasions and whose highest amounts are markedly greater than their average amount per occasion have the greatest risk for injury related to drinking and driving (Gruenewald and Nephew 1994; Gruenewald et al. 1996a,b; Treno and Holder 1997; Treno et al. 1997).

A series of retrospective case control studies have compared the blood alcohol concentration (BAC) levels of people who had experienced trauma (i.e., traffic crashes or other incidents) with the BAC levels of people, usually from the general population, who were not involved in trauma (Cherpitel 1992; Freedland et al. 1993; Fuller 1995; Stoduto et al. 1993; USDHHS 1997; Hurst et al. 1994). In the largest such study, Borkenstein and colleagues (1964) compared the BAC levels of 5,985 drivers involved in traffic crashes with those of 7,590 control drivers in Grand Rapids, Michigan, in 1962 and 1963. For the control sample, police stopped vehicles at preselected sites and times, after which members of the research team requested a voluntary breath sample exclusively for research purposes. A proper statistical analysis of this study (Hurst et al. 1994) found that all levels of BAC were associated with a higher risk of crashes, relative to a BAC of zero, and that the risk of injury increased exponentially with markedly higher BACs.

There are clear reasons why alcohol is related to all kinds of trauma and injury. Even moderate doses of alcohol have cognitive and psychomotor effects that are relevant to the risk of injury, such as effects on reaction time, cognitive processing, coordination, and vigilance (Moskowitz and Robinson 1988; USDHHS 1997; Krüger et al. 1993; Eckardt et al. 1998). Eckardt and colleagues (1998) concluded that the threshold for negative effects on psychomotor tasks is generally found around 0.04 to 0.05 percent BAC.

Driving experience diminishes the adverse impact of alcohol on performance (Preusser et al. 1978). Research has also shown that the relative risk of a fatal crash increases with increasing BAC for each age group but that fatal crash risk for drivers ages 16 to 19 years is higher than the risk for other age groups at all BACs, including zero. This is a result of younger drivers’ lower tolerance for alcohol and their relative inexperience in driving (Mayhew et al. 1985, 1986; Zador 1991).

Dose-response relationships observed in experimental data are not always linear. For example, a recent study with human participants (Lloyd and Rogers 1997) assessed the effects of low doses of alcohol given with a meal and found that a dose of 8 grams of pure alcohol resulted in improved performance on a complex cognitive task in comparison with no alcohol intake, but that 24 grams of alcohol produced impaired performance (Eckardt et al. 1998).

In summary, the evidence indicates that the amount of alcohol consumed per occasion—specifically, the BAC—is the critical feature in determining risk of injury. BACs as low as 0.04 to 0.05 percent may cause psychomotor impairments that lead to increased risk of injury while driving or operating machinery. Alcohol use can be established as a contributing factor in traffic crashes for the following reasons:

- Alcohol is clearly associated with the outcome (i.e., consumption of alcohol increases the risk of being involved in traffic crashes).
- There is a dose-response relationship: the higher the BAC, the higher the chance for injury.
- There is physiological evidence for the relationship.
- Interventions that reduce drinking and driving also reduce alcohol-related traffic crashes. For example, Shults and colleagues (2001) reported in a meta-analysis that random breath testing programs or selective breath testing checkpoints were effective in reducing the mortality of traffic crashes by 18 percent and the number of fatal crashes by 20 percent, in comparison with locations that did not have such programs or checkpoints.

Establishing causality for other forms of alcohol-related injuries is more difficult, even though a strong link may exist. Further research is needed in this area.

## Alcohol and Summary Measures of Health

This article has reviewed research on the observed relationships between alcohol consumption and disease. Many relationships exist, both detrimental and beneficial. This section will review what is known about alcohol and summary measures of health, which are measures that give a general picture of the health of a population, rather than any specific disease risk. One classic summary measure is mortality from all causes. The relationship between average volume of consumption and all-cause mortality in males and females older than 45 is J-shaped, as evidenced by recent meta-analyses (English et al. 1995; Rehm et al. 2001*b*; Gmel et al. 2003*b*). In younger cohorts, a linear relationship prevails—that is, light-to-moderate drinking has no protective effect (Rehm et al. 2001*b*). Indications are that pattern of drinking influences all-cause mortality in all ages as well (Rehm et al. 2001*a*).

All-cause mortality may not, however, be the best summary indicator for measuring alcohol’s impact on health. As indicated above, alcohol use has stronger links to morbidity and disability than to mortality (Murray and Lopez 1997). Thus, a suitable summary indicator (Murray et al. 2000) should integrate data on mortality, morbidity, and disability. Such a summary indicator should also be based on a time measure, such as years of life lost. The Disability Adjusted Life Year (DALY) concept fulfills these requirements ideally. It is a measure that combines years of life lost because of early mortality (i.e., death before the life expectancy in the country with the highest life expectancy worldwide [currently Japan]) with years of life lost to imperfect health (Murray et al. 2000). The relationship between alcohol consumption and DALYs demonstrates that a substantial burden of disease is attributable to alcohol consumption. In 1990 this was estimated as globally higher than the burden of disease attributable to tobacco, even after subtracting the beneficial effects on CHD (Murray and Lopez 1996, 1997).

## Conclusion

Alcohol use is related to a huge health burden in the United States and most countries worldwide, even after discounting for its beneficial effects. In considering this burden, especially for chronic disease, one must keep in mind the limitations of epidemiological studies, which are mostly observational in nature (e.g., cohort studies and case control studies, as described above; also see the sidebar). However, most of the relationships between alcohol use and disease outcomes have also been corroborated by experimental physiological research.

**Methodological Issues Relevant to Studies of Alcohol-Related Morbidity and Mortality**

The relationships between alcohol consumption and disease outcomes have been reported in the alcohol literature based on the best available epidemiological evidence. However, this evidence is to some extent open to question because of the methodological limitations of the studies that produced it, particularly in relation to:

- Measurement of alcohol consumption and theorized exposure
- Measurement of outcome
- Selection of a comparison group
- Design-specific issues. (See also Rehm and Gmel 2003.)

***Measurement of Alcohol Consumption and Theorized Exposure***

For individual-level epidemiological studies, alcohol consumption is usually determined using quantity and frequency (QF) measures. These measures markedly underestimate the volume of drinking in developed countries (e.g., Midanik 1988, 1989; Rehm 1998). Thus, it is problematic to take them at face value—that is, to use them to derive risk relationships that state the risk related to the consumption of a certain volume of alcohol (e.g., grams of pure alcohol). Although no simple solution to this measurement problem exists, alcohol epidemiology has developed better ways to measure alcohol consumption, including measures of both volume of drinking at a certain point in time (e.g., during the last month) and volume accumulated over a longer period, such as the previous 5 years or the entire lifetime. Such measures may be necessary for estimating the associations with chronic diseases such as cancer.

One of these improved measures is the graduated frequency (GF) method, which asks respondents how often they consumed specific quantities, such as 10 to 12 drinks, in a given time period (Greenfield 2000). GF measures have been shown to underestimate volume of alcohol consumed to a smaller degree (Midanik 1994) than quantity and frequency measures and to correlate better with medical outcomes (Rehm et al. 1999*b*). The graduated frequency method also allows researchers to measure irregular heavy drinking patterns as well as volume of drinking at a certain point in time (Greenfield 2000; Rehm 2000). Thus, GF measures seem preferable to the QF measures currently used in medical epidemiology. However, it should be stressed that the method of measuring alcohol consumption used in an epidemiological study should be determined by the theoretically postulated relationship between drinking and disease, and based on the drinking patterns in the culture studied.

The relationship between alcohol consumption and outcome is often based on outcomes assessed at followup, often years after the baseline assessment, with analysis to adjust for the effects of several potential confounder variables such as smoking, age, or socioeconomic status. These procedures must assume that the baseline variables are stable over time, and that they are good indicators of the proposed relationship between alcohol consumption and disease (Sempos et al. 1993). For example, in assessing the relationship between average volume of alcohol consumption and incidence of breast cancer, it must be assumed that heavy consumption persists after baseline and is a good indicator of overall tissue exposure, which is the theoretical risk factor for breast cancer. If an association between two variables, such as heavy drinking and breast cancer, is real, any errors in measuring exposure will “dilute” the strength of the relationship. Therefore, the size of the real effects is often underestimated because of the inaccurate measurement of alcohol consumption at baseline (Clarke et al. 1999). One way to avoid this dilemma is to use measures such as lifetime drinking history in addition to point measures (Russell et al. 1997). The lifetime drinking history is clearly capable of yielding a closer approximation of a theorized lifetime cumulative exposure, which is essential when studying chronic diseases for which accumulated volume of alcohol consumption is the important factor in risk.

For acute outcomes, the usual quantity and frequency measures also do not correspond to the theoretical exposure, which may be BAC or intoxication before the event (e.g., Rossow et al. 2001). Here, objectively measuring BAC or asking directly for degree of intoxication would be preferred options.

Although this sidebar does not review all possible means of measuring alcohol consumption, the examples given should suffice to make the point that the measure of alcohol consumption used in epidemiological studies should reflect the theory underlying the research.

***Measurement of Outcome***

Mortality can usually be measured with relatively little error. Morbidity is more problematic.

On the one hand, many medical epidemiological studies take pains to objectively assess and validate the outcome under consideration. Examples include two coronary heart disease (CHD) studies, the National Health and Nutrition Examination Survey I (NHANES I) followup study on CHD incidence (Sempos et al. 1994), and the World Health Organization MONItoring Trends and Determinants in CArdiovascular Diseases (MONICA) study, a long-term, multinational study on CHD (Tunstall-Pedoe et al. 1999).

On the other hand, studies based on alcohol surveys often measure morbidity by self-assessment, sometimes even including an attribution of causality in the questions respondents are asked (e.g., “Was there ever a time when you felt your alcohol use had a harmful effect on your physical health?”). It is impossible to establish causal relationships with these types of questions (Rehm et al. 1999*a*; Gmel et al. 2000). It is not even possible to establish correlation based on the results of such surveys, beyond the fact that people subjectively believe that there is a relationship between alcohol use and health outcomes. The history of medicine has often shown that such subjective assessment of causality and correlation may not be valid. For instance, women with breast cancer have subjectively attributed their cancer to their number of sexual partners, a causal relation that has no objective basis (Geyer 2000). Similarly, people may attribute health and other problems to alcohol in cases where there is no real relationship. As a consequence, future studies linking alcohol and disease should try to combine the rigorous objective assessment of outcomes used in medical epidemiology with state-of-the-art measurement of alcohol epidemiology.

***Selection of a Comparison Group***

Studies of alcohol-related health outcomes often use abstainers as a comparison group. Abstainers, however, are not a homogeneous group, at least in developed countries. Many people stop drinking because they have become sick, thus increasing the relationship between abstention and disease (Shaper 1990). Studies of disease risk that compare different levels of drinking and no drinking may overestimate the protective effect of light-to-moderate drinking if abstainers are not separated into lifetime abstainers and former drinkers (Rehm et al. 2001). For example, when the relationship between alcohol use and disease forms a J-shaped curve (see the figure in the article), the increased risk observed for abstainers may reflect the fact that some former drinkers became current abstainers because of health problems. Even if this distinction is made, interpreting risk differences for current drinkers compared with lifetime abstainers may be difficult, because lifetime abstainers in many societies differ from the general population in several other ways, such as diet, religion, or socioeconomic status (e.g., Bondy and Rehm 1998). Because long-term studies cannot manipulate abstention status experimentally, there is no final way to determine the causal influence of alcohol consumption. However, statistical control of potential confounders is possible.

***Design-Specific Issues***

Of the two major categories of research designs, individual-level studies produce results that deserve greater attention than the results of aggregate-level, or ecological, studies, especially when the individual-level results are corroborated by evidence from physiological experiments. However, specific aggregate-level examples with interventions, such as the study of the Gorbachev anti-alcohol campaign (described in the article), play an important role in alcohol epidemiology.

The Gorbachev campaign is an exception for several reasons: It was a natural experiment with a purposeful change in one variable; it affected a large outcome category (mortality from all causes); its effects cannot be explained by changes in the system used to document causes of death or other changes; and it had very large effects. Other ecological analyses in the alcohol field tend to show correlations over time or countries, which can hardly be interpreted because confounding cannot be excluded.

Generally, however, more value is placed on individual-level analysis than on ecological analysis. The two individual-level designs most often used are case control and cohort designs. Cohort studies may be especially problematic in alcohol epidemiology. First, many large-scale cohorts are selected in a way to minimize dropouts (i.e., based on their members’ availability for repeated followups). As a result, nurses, doctors, and other health professionals often make up these cohorts. Selecting cohorts on this basis reduces the variation within cohorts with respect to alcohol consumption. As a consequence, the effects of certain drinking patterns cannot be explored in such cohorts. Some cohort studies—such as the American Cancer Society’s Cancer Prevention Study II, a nonrepresentative general population study based on the American Cancer Society’s members and friends (Thun et al. 1997)—make use of a wider range of drinking styles. These cohort studies, however, do not include some patterns of drinking that are customary in other parts of the world or in other U.S. population subgroups (e.g., Native Americans, the homeless, and other disadvantaged groups), and thus the effects of these drinking patterns cannot be studied. These limitations of cohort studies for use in alcohol epidemiology are purely practical and do not change the theoretical advantages of cohorts over case control studies. However, if the relevant behavior is not present in a cohort, no conclusions can be drawn. In sum, in many cohorts studied in medical epidemiology, certain patterns are either not represented or represented in a manner that does not allow for analysis. Thus, future cohort studies should include people with more varied consumption patterns, such as irregular heavy drinking.

*—Jürgen Rehm, Gerhard Gmel, Christopher T. Sempos, and Maurizio Trevisan*

ReferencesBondySRehmJThe interplay of drinking patterns and other determinants of healthDrug and Alcohol Review1739941219981620350710.1080/09595239800187241ClarkeRShipleyMLewingtonSUnderestimation of risk associations due to regression dilution in long-term follow-up of prospective studiesAmerican Journal of Epidemiology15034135319991045381010.1093/oxfordjournals.aje.a010013GeyerSThe role of social and psychosocial factors in the development and course of cancerWiener Klinische Wochenschrift112986994200011190714GmelGRehmJRoomRGreenfieldTKDimensions of alcohol-related social harm in survey researchJournal of Substance Abuse1211313820001128846610.1016/s0899-3289(00)00044-4GreenfieldTKWays of measuring drinking patterns and the difference they make: Experience with graduated frequenciesJournal of Substance Abuse12335020001128847310.1016/s0899-3289(00)00039-0MidanikLTValidity of self-reported alcohol use: A literature review and assessmentBritish Journal of Addiction83101910291988306641810.1111/j.1360-0443.1988.tb00526.xMidanikLTPerspectives on the validity of self-reported alcohol useBritish Journal of Addiction84141914231989269274110.1111/j.1360-0443.1989.tb03920.xMidanikLTComparing usual quantity/frequency and graduated frequency scales to assess yearly alcohol consumption: Results from the 1990 U.S. National Alcohol SurveyAddiction894074121994802549310.1111/j.1360-0443.1994.tb00914.xRehmJMeasuring quantity, frequency and volume of drinkingAlcoholism: Clinical and Experimental Research224S14S199810.1097/00000374-199802001-000029603301RehmJRe: Alcohol intake assessment: The sober factsAmerican Journal of Epidemiology151436438200010.1093/oxfordjournals.aje.a01022510695604RehmJGmelGAlcohol consumption and total mortality/ morbidity: Definitions and methodological implicationsBest Practices & Research Clinical Gastroenterology17497505200310.1016/s1521-6918(03)00032-512828951RehmJFrickUBondySA reliability and validity analysis of an alcohol-related harm scale for surveysJournal of Studies on Alcohol602032081999a1009195810.15288/jsa.1999.60.203RehmJGreenfieldTKWalshGAssessment methods for alcohol consumption, prevalence of high risk drinking and harm: A sensitivity analysisInternational Journal of Epidemiology282192241999b1034268210.1093/ije/28.2.219RehmJGutjahrEGmelGAlcohol and all-cause mortality: A pooled analysisContemporary Drug Problems283373612001RossowIPernanenKRehmJAccidents, suicides and violenceKlingemannHGmelGMapping the Social Consequences of Alcohol ConsumptionDordrecht, NetherlandsKluwer Academic Publishers200193112RussellMMarshallJRTrevisanMTest-retest reliability of the Cognitive Lifetime Drinking HistoryAmerican Journal of Epidemiology1469759811997940034010.1093/oxfordjournals.aje.a009225SemposCTFlegalKMJohnsonCLIssues in the long-term evaluation of diet in longitudinal studiesJournal of Nutrition123405412199310.1093/jn/123.suppl_2.4068429395SemposCTLookerACGillumRFMakucDMBody iron stores and the risk of coronary heart diseaseNew England Journal of Medicine330111911241994799340510.1056/NEJM199404213301604ShaperAGAlcohol and mortality: A review of prospective studiesBritish Journal of Addiction858378471990220445410.1111/j.1360-0443.1990.tb03710.xThunMJPetoRLopezADAlcohol consumption and mortality among middle-aged and elderly U.S. adultsNew England Journal of Medicine337170517141997939269510.1056/NEJM199712113372401Tunstall-PedoeHKuulasmaaKMähönenMContribution of trends in survival and coronary-event rates to changes in coronary heart disease mortality: 10-year results from 37 WHO MONICA Project populationsLancet3531547155719991033425210.1016/s0140-6736(99)04021-0

Much of the alcohol-related health burden could be avoided by initiating or strengthening policy measures proven to be effective in reducing alcohol use and related problems, such as taxing consumption, restricting access to alcohol, and random breath testing (Edwards et al. 1994). Some of these measures (e.g., taxation, restricting access) have been shown to reduce the social harm caused by alcohol consumption as well. Given the size of the burden of disease related to alcohol use and the availability of effective countermeasures, there seems to be no justification for continuing the status quo.

## Footnotes

1 In the United States, a drink is considered to be 0.5 ounces (oz) or 15 grams of alcohol, which is equivalent to 12 oz (355 milliliters [mL]) of beer, 5 oz (148 mL) of wine, or 1.5 oz (44 mL) of 80-proof distilled spirits (U.S. Department of Health and Human Services [USDHHS] 2000).
